# Fully convolutional network for rice seedling and weed image segmentation at the seedling stage in paddy fields

**DOI:** 10.1371/journal.pone.0215676

**Published:** 2019-04-18

**Authors:** Xu Ma, Xiangwu Deng, Long Qi, Yu Jiang, Hongwei Li, Yuwei Wang, Xupo Xing

**Affiliations:** College of Engineering, South China Agricultural University, Guangzhou, China; Newcastle University, UNITED KINGDOM

## Abstract

To reduce the cost of production and the pollution of the environment that is due to the overapplication of herbicide in paddy fields, the location information of rice seedlings and weeds must be detected in site-specific weed management (SSWM). With the development of deep learning, a semantic segmentation method with the SegNet that is based on fully convolutional network (FCN) was proposed. In this paper, RGB color images of seedling rice were captured in paddy field, and ground truth (GT) images were obtained by manually labeled the pixels in the RGB images with three separate categories, namely, rice seedlings, background, and weeds. The class weight coefficients were calculated to solve the problem of the unbalance of the number of the classification category. GT images and RGB images were used for data training and data testing. Eighty percent of the samples were randomly selected as the training dataset and 20% of samples were used as the test dataset. The proposed method was compared with a classical semantic segmentation model, namely, FCN, and U-Net models. The average accuracy rate of the SegNet method was 92.7%, whereas the average accuracy rates of the FCN and U-Net methods were 89.5% and 70.8%, respectively. The proposed SegNet method realized higher classification accuracy and could effectively classify the pixels of rice seedlings, background, and weeds in the paddy field images and acquire the positions of their regions.

## Introduction

Rice is one of the major global food crops that feeds over 65% of the Chinese [1]; however, weeds in farmland impede the growth of crops. Weeds decrease rice production by competing for moisture, nutrients, and light in paddy fields [2]. In traditional agriculture, the main weeding method of spraying chemical herbicides extensively without distinguishing between crops and weeds not only results in the waste of herbicide and labor forces but also causes environmental pollution and health hazards for humans [3]. Precise pesticide spraying via site-specific weed management (SSWM) in smart farming can reduce the pesticide consumption by approximately 40–60%, thereby reducing the environmental pollution and increasing the economic profits [4]. For realizing these benefits, identifying weeds and their positions accurately and automatically is the foundation of site-specific spraying.

The site-specific spraying of herbicides requires the generation of a weed cover map. However, in the past, academics only utilized the projection method with a binary image to calculate the weedy areas [5, 6]. With the application of plant protection using unmanned aerial vehicle (UAVs), weed target detection that is based on UAVs has attracted increasing attention. Weed overlays were acquired via the projection calculation method from spectral images at 30, 60 and 90 meters in maize and sunflower fields [7–9]. However, it remains difficult to obtain accurate information on weed areas on a small scale and to distinguish between crops and weeds.

As a branch of machine learning, deep learning has been widely applied in various fields and has developed into a powerful method for image classification [10, 11] and object detection [12]. The object detection framework of regions with convolutional neural networks (R-CNN) [13] and Fast R-CNN [14] with selective search with region proposal [15] have produced breakthroughs. The region proposal network produces fewer and higher-precision proposed regions in Faster R-CNN [16]. In addition to these object detection methods, which are based on region classification, the bonding box regression method has been used by POLO [17] and single-shot multi-box detector [18]. This technique overcomes the lower location precision of the method for region classification and can balance efficiency and precision in object detection.

Object detection algorithms that are based on deep learning have realized tremendous improvements in accuracy and speed compared to traditional detection algorithms and exhibit higher feature extraction performance due to the use of convolutional neural network (CNN). Typically, these methods perform well in object detection; however, they require a bounding box that tightly surrounds the object of interest. Because rice seedlings and weeds do not have definite boundaries and can lead to partial occlusions in the limited space, it will be challenging to clearly delineate boundaries of rice seedlings and weeds. The method might not be viable for object detection at the seedling stage. Moreover, the morphological diversity of the growth stages of weeds creates unprecedented challenges for using object detection with a bounding box.

The goal of image semantic segmentation was to obtain the categorized results of each pixel at corresponding position. The method with patch-level is to take an image tile at the center of some pixel point, and the features of image patches were used as the sample set to train the classifiers [19]. However, the patch-level method is time consuming. Additionally, one of the drawbacks of the method was that the performance of algorithm was affected and limited by the image patch, where the model cannot be performed on the base of larger context information. The method with pixel-level is based on fully convolutional network (FCN) [20] was introduced for obtaining the position of every pixel, and the features of pixel points were used as the sample set to train the classifiers. What is more, it solved the problem with semantic segmentation of patch-level efficiently and effectively. FCN can accept input image with any size and retain the pixel spatial information in the original input image, which can classify each pixel on the feature map. It provides the potential to generate the cover map of weeds in SSWM applications.

The FCN method overcomes target occlusion and realizes substantial improvements in image segmentation performance [21, 22] via per-pixel classification. Deep learning has been widely used in many fields of agriculture [23]. The FCN method was used to generate a weed cover map with UAV imagery [24–26] over a rice field, which demonstrated that this method is fast and can be used in the semantic segmentation of weeds. However, UAV imaging mainly about large-scale images, which do not meet the requirements for small-area detection with weeds on small scales in paddy fields.

SegNet, first put forward by Cambridge [27], is a deep full convolutional neural network used in image segmentation. SegNet uses the symmetric codec structure and index structure of max pooling to obtain multi-scale information, which has lower computational cost and higher precision than FCN. Because of using structure of dilated convolution, DeepLab [28] and PSPNet [29] have better accuracy. But compared to them, SegNet has simpler structure and its computing speed is faster. Semantic segmentation of rice seedlings and weeds is a simple classification in three categories, and SegNet is well-suited for processing the segmentation of small irregular-shaped rice seedlings and weeds in the paddy field.

The novelty and contributions of the paper is that we proposed a robust and fast image segmentation method for rice seedlings and weeds at the seedling stage in the paddy field based on SegNet, where rice seedlings and weeds shading each other. The main objectives of this study were to (1) propose a semantic segmentation method that is based on the encoder and decoder parts and (2) analyze and compare the performance of proposed method with those of a classical semantic segmentation model, namely, FCN, and U-Net models.

## Material and methods

### Dataset

#### Image capture

Sagittaria trifolia, which is a perennial troublesome weed [30], mainly grows in paddy fields and reduces rice production dramatically. Sagittaria trifolia becomes widespread and its damage has been increasing year after year [31]. Thus, research on sagittaria trifolia in paddy fields is necessary.

**Fig 1:**
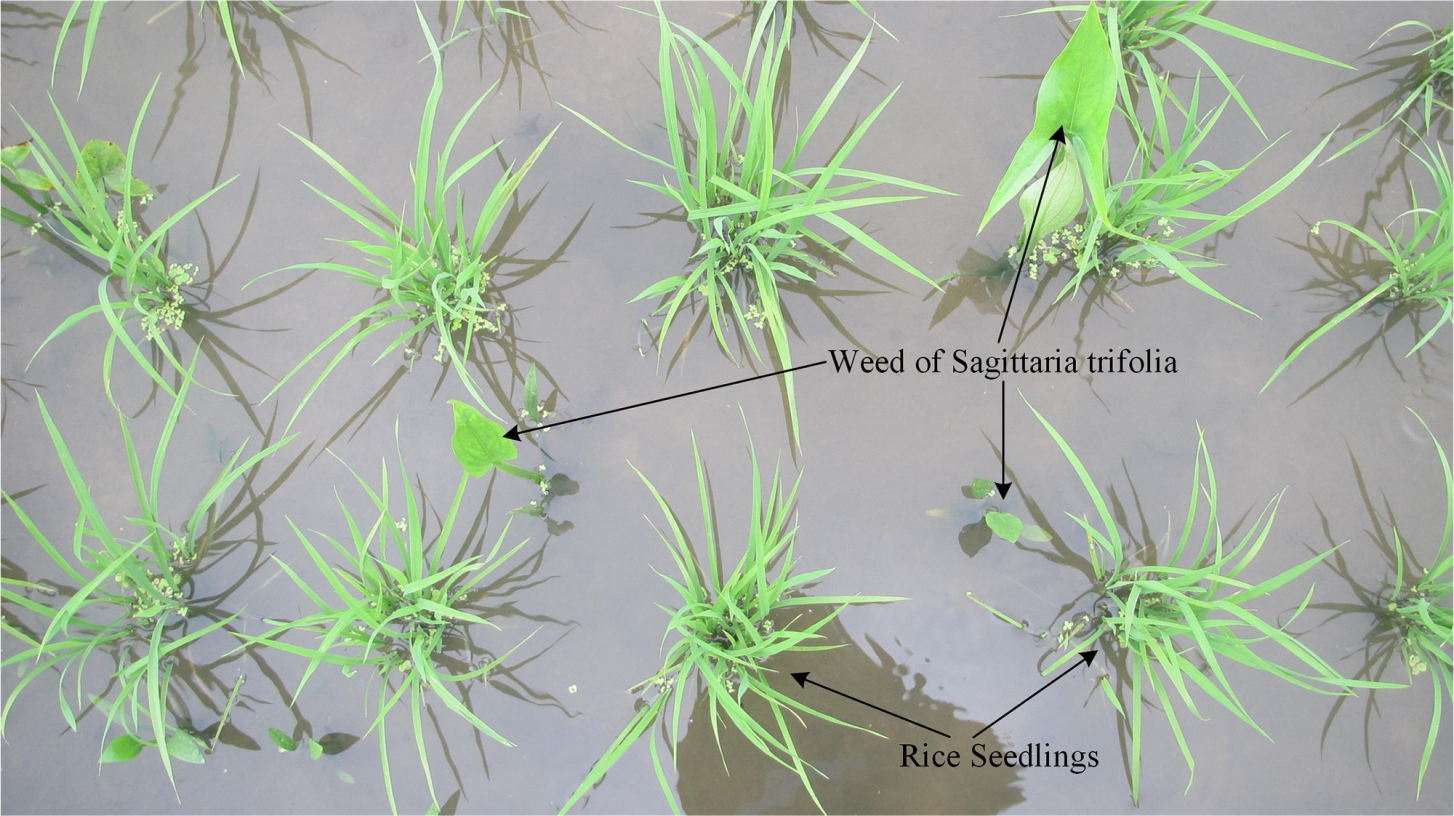
Rice seedlings and weeds images in the paddy field.

Images of weeds in paddy fields were captured using a Canon IXUS 1000 HS (EF-S 36–360 mm f/3.4–5.6 IS STM) camera on April 13, 2018, which was approximately 20 days after the rice seedlings had been transplanted via mechanization. The row spacing of the rice seedlings was 300 mm and the plant spacing was 140–160 mm. The image was of size 3648×2048 pixels and included many rice seedlings and weeds in the scene and the acquisition format was color RGB images. The paddy fields were located in Jiangmen, Guangdong province (22°30′26.97″N, 113°05′45.54″E). The camera was 800–1200 mm above the water surface of the fields during image capture. The images were selected in the paddy fields and all weeds were in early growth stages. Fig 1 shows sample images of the rice seedlings and weeds. A total of 28 images were captured.

#### Data collection

**Fig 2:**
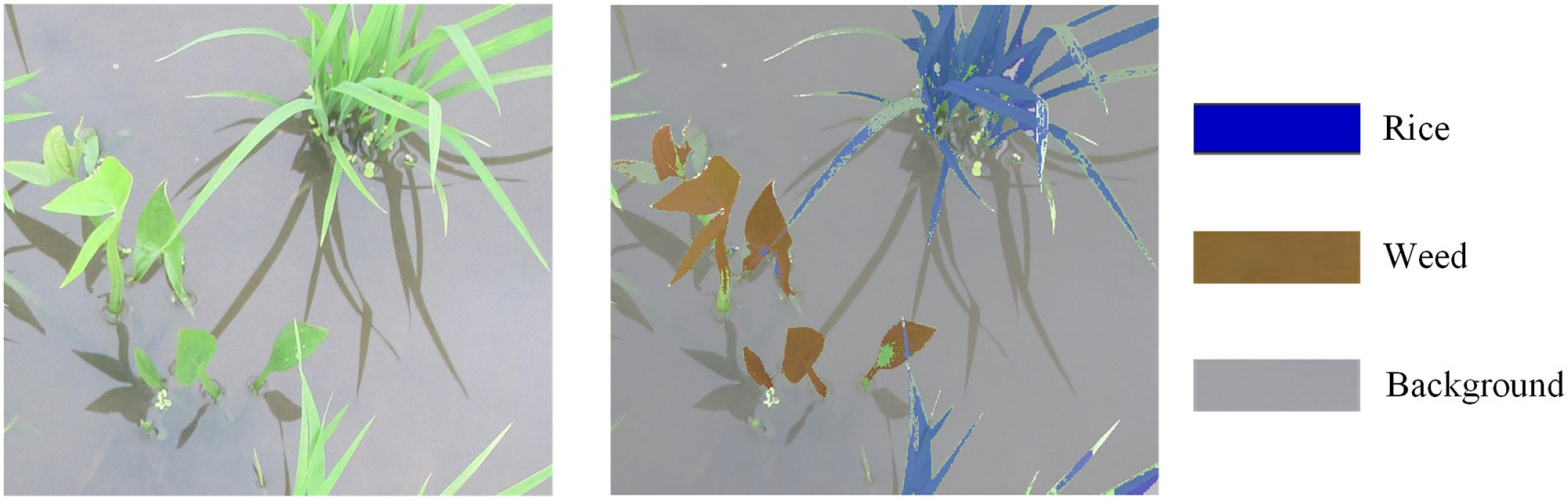
Image-label example. (a) original image and (b) the corresponding GT labels.

The original image is 3648×2048, which is much larger than the input image size in [20, 27] and could lead to the exhaustion of the GPU memory. Therefore, each image was split into two lines and four columns, which was divided into 8 tiles of size 912×1024 pixels and the tiles number was 224. The ground truth (GT) images were obtained by manually tagged the semantic labels of each pixel on the original RGB images with three separate categories (Fig 2): rice seedlings, weeds, and background (including reflection and water), which used to train the model with the training dataset and calculate the performance with the test dataset. The data sample included GT images and RGB images. Eighty percent of the samples were randomly selected as the training dataset and 20% of samples were used as the test dataset.

#### Class weight coefficients

As shown in Table 1, the pixel number of weed, rice seedling, and background accounted for 5.028%, 11.517%, and 83.455% of the total pixel number with image dataset respectively, and the number of pixels of with three categories were imbalance. Take the pixel number of rice seedling as the median frequency class weights, we can ascertain other class weight coefficients of weed and background, and realize the balance with the number of the classification category. For example, the large pixel number of background sample should be set to the small weight coefficient, and that of weed sample should be set to a big one. The calculation method is expressed in (Eq 1).

$$w_{j}=\frac{\sum_{i}\frac{N_{\text{i}}}{3}}{\sum_{i}N_{ij}}$$ (1)

where *w*_j_ denotes the class weight coefficient, *N*_ij_ denotes the number of pixels in image *i* that belong to class *j*, and *N*_i_ denotes the total number of pixels that belong to the *i*th image.

**Table 1:** Number of pixels with classes and the class weight coefficients.

Pixel type	Percentage/%	Class weight coefficients
Weed	5.028	2.280
Rice seedling	11.517	1.000
Background	83.455	0.138

### Methods

#### Network architecture of SegNet

**Fig 3:**
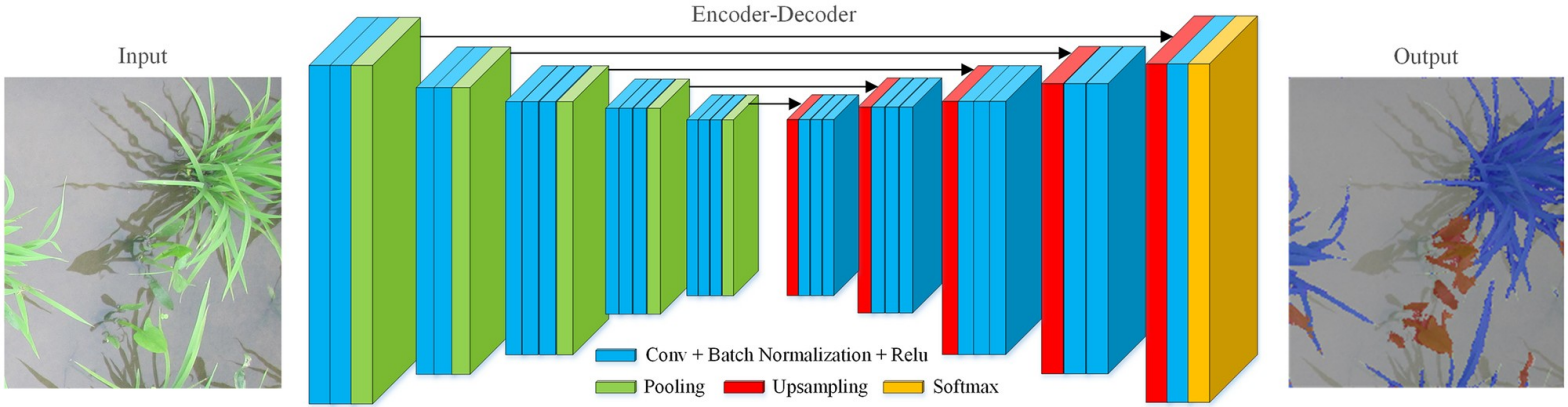
Network architecture of SegNet.

The framework of SegNet was introduced in 2016 [27], which realized pixel-level classification via end-to-end training. SegNet was proposed as the underlying architecture for semantic segmentation with rice seedling and weed images at the seedling stage in a paddy field, which includes an encoder, a decoder, and a softmax classifier. The encoder included the first 13 convolutional layers of the pretrained VGG16 [32] and the structure of decoder was symmetric to that of the encoder. The structure of the encoder and decoder ensured that the input size of SegNet was the same as the output size. Each encoder and decoder unit is comprised of five main sections. Each section in the encoder contains a convolutional layer (Conv), a batch normalization (BN), and the activation function of a rectified linear unit (ReLU). The ReLU is used to activate the output of BN and BN processes the output of Conv. The network architecture of SegNet is illustrated in Fig 3. The parameters of the Convs in SegNet are listed in Table 2.

**Table 2:** Encoder and decoder parameters of SegNet.

Encoder	Layer type	Feature size	Number of features	Decoder	Layer type	Feature size	Number of features
**1**	Conv1_1	3×3	64	**5**	Conv5_3	3×3	512
	Conv1_2	3×3	64		Conv5_2	3×3	512
**2**	Conv2_1	3×3	128		Conv5_1	3×3	512
	Conv2_2	3×3	128	**4**	Conv4_3	3×3	512
**3**	Conv3_1	3×3	256		Conv4_2	3×3	512
	Conv3_2	3×3	256		Conv4_1	3×3	512
	Conv3_3	3×3	256	**3**	Conv3_3	3×3	256
**4**	Conv4_1	3×3	512		Conv3_2	3×3	256
	Conv4_2	3×3	512		Conv3_1	3×3	256
	Conv4_3	3×3	512	**2**	Conv2_2	3×3	128
**5**	Conv5_1	3×3	512		Conv2_1	3×3	128
	Conv5_2	3×3	512	**1**	Conv1_2	3×3	64
	Conv5_3	3×3	512		Conv1_1	3×3	64

For extracting efficient features through the Convs and the pooling layers in the encoder, the dimension of the features increases as the Convs deepen. The size of the feature map decreases continuously and the pixel value decreases with the size of the pooling layer in the encoder and the size of the feature map can be restored via deconvolution and upsampling in the decoder. The encoder stores the position information of the pixel in the maxpooling process, which ensures the edge completeness via upsampling in the decoder. The missing pixel values are filled via deconvolution and upsampling.

#### Transfer learning

As the main structure of SegNet was composed mainly of the pretrained CNN model of VGG16 in the encoder, the model of SegNet was trained via transfer learning. Transfer learning [33] is a new machine learning method that applies the knowledge from related but different domains to target domains, which aiming to solve the problems that there are few or even not any labeled data in target domains. To the small sample image data sets in this article, transfer learning with the well trained CNN model of VGG16 in image classification can migrate to pixels classify model.

#### Upsampling and deconvolution

**Fig 4:**
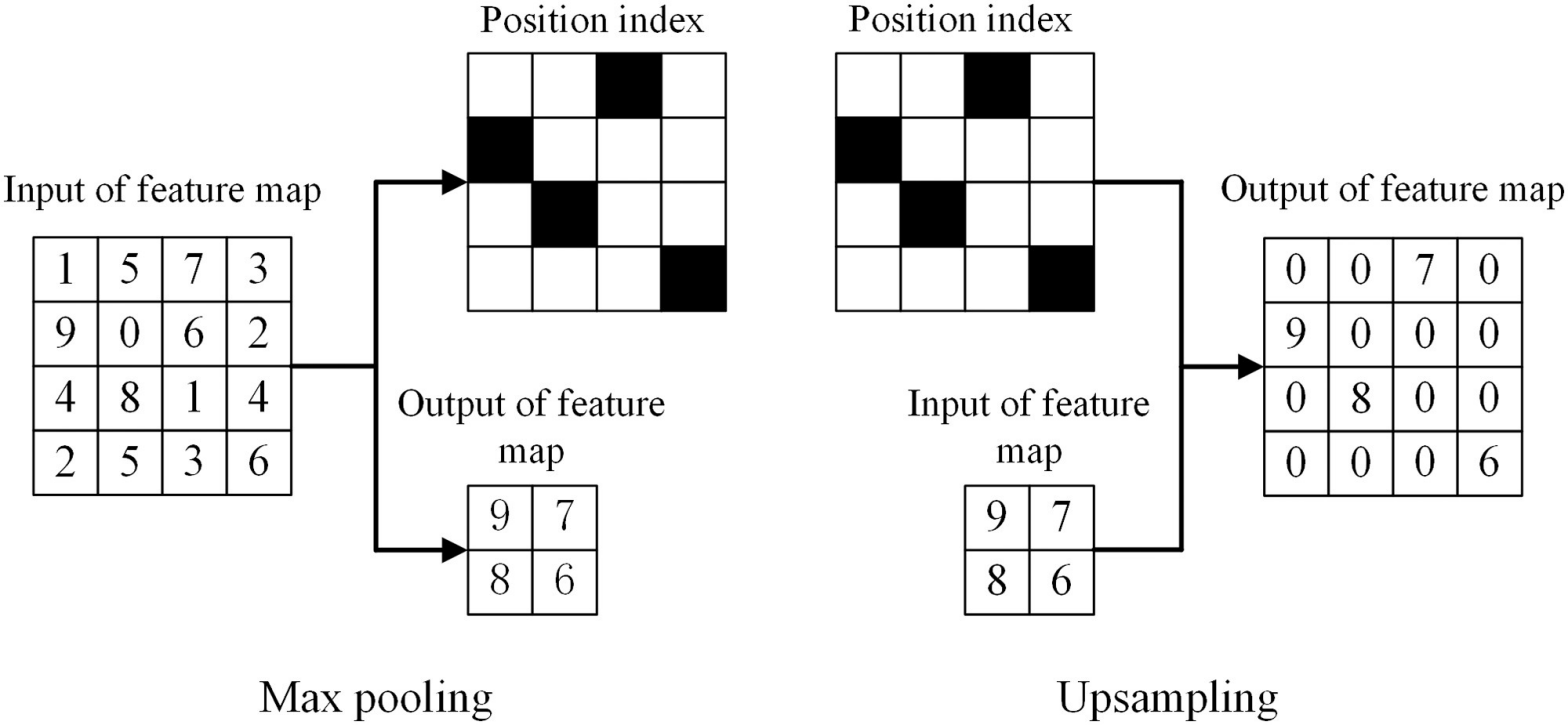
Pooling and upsampling.

Each pixel that corresponds to the rice seedlings, backgrounds, and weeds in the images was classified based on SegNet. As shown in Fig 4 as the flow diagram on the left, data were lost and the input of the feature map is half the size of the output of a maxpooling operation, which leads to the progressive diminishment of the marginal information on the rice seedlings and weeds and to this information being obscured by the process of each section of the encoder. In the process of each section of the decoder, upsampling and deconvolution were utilized to preserve the size of the output image of SegNet and the sharp edges of the rice seedlings and weeds. As shown in Fig 4 as the flow diagram on the right, data that were lost during the pooling process were replaced during the deconvolution process. The position index was saved to ensure the unabridged edges, which was the relative position of the maximum value in the pooling. The 4×4 feature map can generate two related outputs after maxpooling: a 2×2 feature map and a 4×4 position index. This information subsequently served as input during the process of upsampling, where the remaining numerical values of the position index were filled by 0 s. The deconvolutional layer was the same as the convolutional layer, which was after the upsampling layer.

### Evaluation metrics

The pixel accuracy (PA) is the number of pixels that are correctly classified to an individual class as a percentage of the total number of pixels. The mean pixel accuracy (MPA) is an extension of PA, which refers to the percentage of correctly classified pixels for each category and is calculated as the average of all PAs over all the classes. The intersection over union (MIoU) is the standard measure of the semantic segmentation, which is calculated as the union of the ground truth and predicts the segmentation according to the pixel class. The frequency-weighted intersection over union (FWIoU) is an extension of MIoU in which weights are assigned according to the frequency of each class. In addition, the speed of image processing is the key index for practical use. Hence, this paper will consider MPA, MIoU, FWIoU and speed in the performance analysis of the semantic segmentation model. The calculation process is expressed in Eqs 2 to 5.

PA=∑i=0kpii∑i=0k∑j=0kpij (2)

MPA=1k+1∑i=0kpii∑j=0kpij (3)

$$\text{MIoU}=\frac{1}{k+1}\sum_{i=0}^{k}\frac{p_{ii}}{\sum_{j=0}^{k}p_{ij}+\sum_{j=0}^{k}p_{ji}-p_{ii}}$$ (4)

$$\text{FWIoU}=\frac{1}{\sum_{i=0}^{k}\sum_{j=0}^{k}p_{ij}}\sum_{i=0}^{k}\frac{p_{ii}}{\sum_{j=0}^{k}p_{ij}+\sum_{j=0}^{k}p_{ji}-p_{ii}}$$ (5)

where *k* represents the class of rice seedlings, weeds, and background and *k* is equal to 2 in this experiment; *i* represents the real class; *j* represents the predicted class; *P*_ii_ represents the number of true positives, namely, the number of pixels for which the real class and the predicted class are the same; *P*_ij_ represents the number of false positives, namely, the number of pixels that were misclassified; and *P*_ji_ represents the number of false negatives, namely, the number of pixels that were correctly classified.

## Results and discussion

### Comparison models

#### FCN

The network structure of FCN [20] is a classical model with semantic segmentation, which was improved based on the CNN of AlexNet and was applied to natural images for pixel-level classification. The framework of AlexNet is divided into two parts: the front part and the latter part. The front part, which includes convolutional layers and pooling layers, is used to perform the feature extraction and the latter part consists of three fully connected layers. FCN includes the feature extraction part of AlexNet and the fully connected layers are converted into convolutional layers. The output feature map of the last convolutional layer performs upsampling by deconvolution via the bilinear interpolation algorithm, which leads to output and input images that are of the same size in the FCN.

#### U-Net

The framework of U-Net [34] was proposed by Ronneberger in 2015 and performs well on biomedical images. U-Net has two sections: a shrinking structure and an expanding structure. Unlike the principles of FCN for the convolution and deconvolution operations of feature maps, the shrinking structure constantly extracts the context information and the expanding structure combines the feature maps, where the lost edge information is constantly replenished. Then, more accurate predictions of the pixel points on the edge can be obtained.

### Experiments on SegNet

#### Training parameters

The pixels of each image were labeled and the model was trained and validated with the test dataset on the MATLAB 2017b software. The computer was configured with 16 G memory, an Intel@Core(TM) i7-8700K CPU @ 3.70 GHz ×6 processor memory and a VIDIA GeForce GTX 1080Ti GPU.

The main parameters for this SegNet model were set as follows: the number of iterations was 30, the batch size was 1, the weight learning speed was 0.001, the momentum was 0.9, the regularization model was L2, and the regularization parameter, namely, λ, was 0.0005. The stochastic gradient descent is adjusted as needed during the neural network training process.

#### Feature map visualization

**Fig 5:**
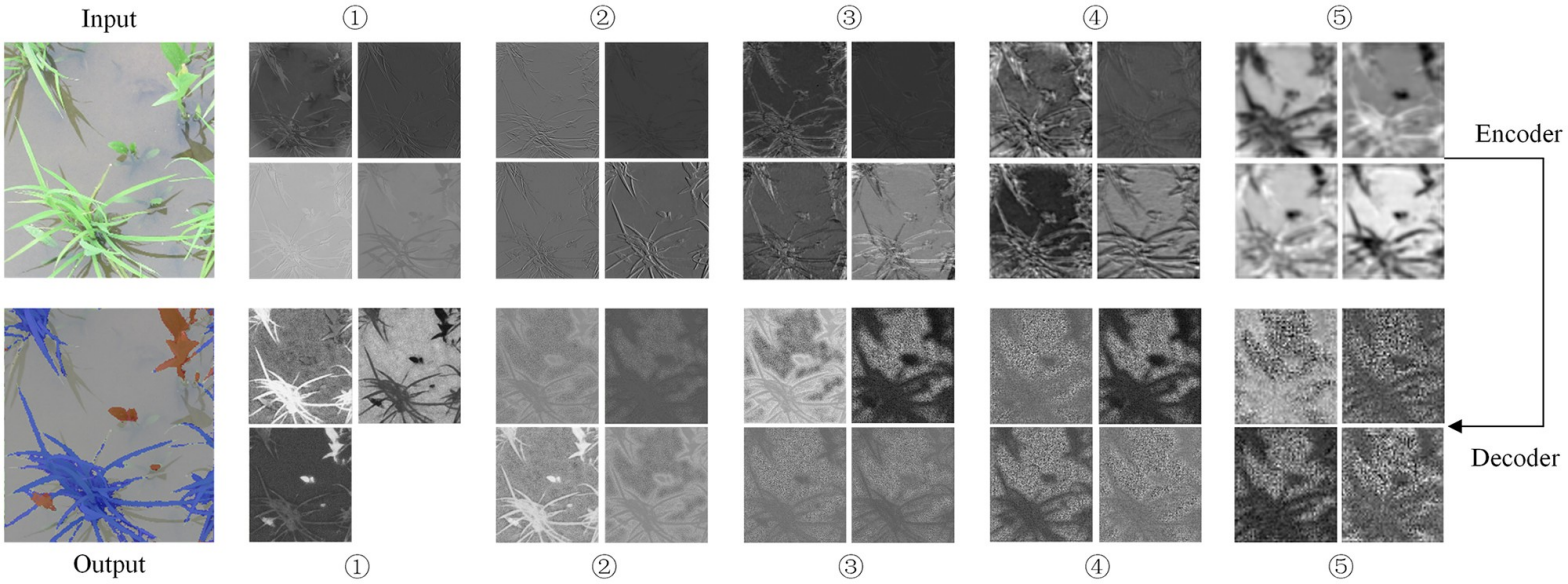
Feature map visualization of the convolutional layer.

The features were automatically extracted in the convolutional layer from the stack-based feature maps and our method for feature extraction from pixel-level data was utilized. The SegNet was established based on the encoder and the decoder. To better understand what the Convs have learned and the learning processes in the encoder and the decoder, the feature map of the last Conv with each encoder and decoder section is shown in Fig 5, which was obtained via a visualization method. Four feature maps of the last Conv were selected as representatives of each section in the encoder and the decoder and because in the input channel of Conv1_1 in the encoder, the three color components of the RGB image were the same as in the output channel of Conv1_1 in the decoder, the size of the feature map of Conv1_1 in the decoder was only 3. The feature extraction of SegNet was based on CNN of VGG16, where the feature map size become small and the data structure become sparser by the operation with the pooling layer and Convs. The feature map becomes increasingly abstract and vague as the Convs deepen in the encoder. Because of the deeper the encoder, the feature map will receive more operation with the pooling layer and Convs. On the one hand, the reversible process of feature mapping is performed in the decoder via deconvolution and upsampling. So, the output image of the SegNet was the same as the input size.

#### Comparison of the results

**Fig 6:**
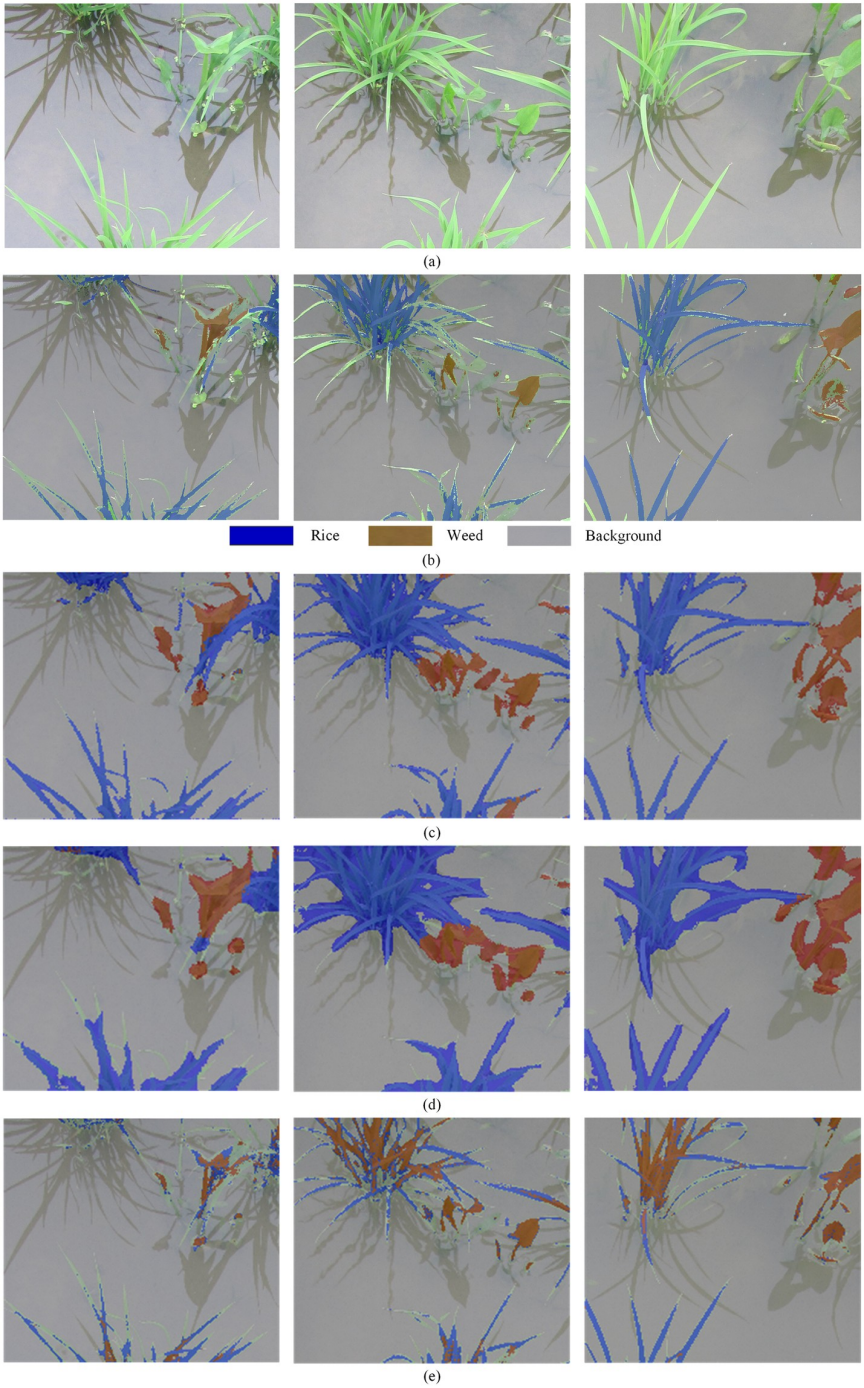
Performance comparison on test images. (a) original images; (b) ground truth; (c) output by our method; (d) output by FCN; and (e) output by U-Net.

In the equivalent training and testing environments and with the same test and training datasets, SegNet outperformed the FCN and U-Net models. The FCN model could accurately detect the pixels of rice seedlings, weeds, and background. The bilinear interpolation algorithm was used in the processes of deconvolution and upsampling with FCN, which has the advantages of fewer calculations and easier implementation. However, it easily overlooks the section details of the border, the edges are thickened and marginal haziness is observed. The network structure of SegNet was simplified into the encoder and decoder. The position information of maxpooling was stored in the encoder and the missing pieces were filled via deconvolution and upsampling in the decoder. However, in contrast to the bilinear interpolation algorithm, the location property of the characteristic points was preserved and the BN layer was introduced into SegNet; hence, SegNet yielded semantic information that was closer to the GT and the edge detection precision was higher than that of FCN. The target detection performance of the U-Net model was superior; however, the misclassification rates of pixels that corresponded to rice seedlings and weeds were higher. Experiment results for SegNet demonstrated the satisfactory segmentation performance for pixel classification of rice seedlings, weeds and background, as shown in Fig 6.

The experimental results for the three semantic segmentation models on the test datasets are listed in Table 3. Comparing the evaluation metrics of the three approaches, SegNet typically outperformed FCN and U-Net on the three evaluation indices: MPA, MIoU and FWIoU. Since PA was used to estimate the percentage of correctly classified pixels, regardless of the class, MPA was an important performance metric for the semantic segmentation network. The proposed SegNet method realized higher classification accuracy, with an average accuracy rate of 92.7%, followed by the FCN and U-Net methods, with average accuracy values of 89.5% and 70.8%, respectively. However, the structure of SegNet was more complex and deeper compared to the other two models and required longer time for the image processing (0.604 s vs. 0.148 s and 0.331 s with an image size of 912×1024 pixels), which meets the application requirements in agriculture.

**Table 3:** Results of SegNet, FCN and U-Net.

Approach	MPA	MIoU	FWIoU	Speed
**SegNet**	0.927	0.618	0.844	0.604 s
**FCN**	0.895	0.538	0.759	0.148 s
**U-Net**	0.708	0.530	0.831	0.331 s

Evaluation of the pixel classification models in terms of precision was carried out by examining the confusion matrix, which is presented in Table 4. Compared with the existing pixelwise classification methods of FCN and U-Net, the proposed SegNet method realized higher classification accuracy. The accuracy rates for rice, background and weed pixelwise classification with the SegNet method were 93.6%, 90.7% and 93.9%, respectively. On rice and weed pixelwise classification, substantially more accurate results were obtained compared to the other two approaches. The accuracy rate of background pixelwise classification with U-Net was 97.7%, which was higher than those of the other approaches. For the rice and weed pixelwise classifications, the U-Net model yielded low accuracy. The results demonstrate that the proposed pixelwise classification method, which is based on SegNet, could effectively classify the rice, background, and weeds in paddy field images. The symmetric structures of the encoder and decoder were established in SegNet, which were used to extract the multiscale feature and increase the accuracy of feature extraction. SegNet was well-suited for processing the pixel classification of images of tiny and abnormally shaped rice seedlings and weeds in the paddy field.

**Table 4:** Comparison of the SegNet, FCN and U-Net approaches.

Approach	GT/Predicted Class	Rice	Background	Weed
**SegNet**	Rice	**0.936**	0.015	0.049
	Background	0.061	**0.907**	0.032
	Weed	0.042	0.019	**0.939**
**FCN**	Rice	**0.921**	0.022	0.056
	Background	0.120	**0.834**	0.046
	Weed	0.053	0.018	**0.929**
**U-Net**	Rice	**0.462**	0.182	0.356
	Background	0.014	**0.977**	0.010
	Weed	0.173	0.143	**0.685**

## Conclusions

This paper proposed a semantic segmentation method that is based on fully convolutional network with the SegNet model, which can extract the features from initial RGB images directly and classify and recognize the pixels that correspond to rice, background, and weeds in paddy field images. The proposed method is compared with a classic semantic segmentation model, namely, FCN, and U-Net models in terms of performance. The symmetric structure of encoding and decoding was established in SegNet, which was used to extract the multiscale features and improve the accuracy of feature extraction. SegNet was well-suited for processing the pixel classification of images of tiny and abnormally shaped rice seedlings and weeds in paddy fields. The proposed SegNet method realized higher classification accuracy. The average accuracy rate of the SegNet method was 92.7%, whereas the average accuracies of the FCN and U-Net methods were 89.5% and 70.8%. The proposed pixelwise classification method, which is based on fully convolutional neural networks, could effectively classify the rice, background and weeds in paddy field images. At the same time, this method could perform pixel classification of RGB images in real time to meet the application requirements.
